# Analysis of three strategies to increase screening coverage for cervical cancer in the general population of women aged 60 to 70 years: the CRICERVA study

**DOI:** 10.1186/1472-6874-14-86

**Published:** 2014-07-16

**Authors:** Amelia Acera, Josep Maria Manresa, Diego Rodriguez, Ana Rodriguez, Josep Maria Bonet, Norman Sanchez, Pablo Hidalgo, Pilar Soteras, Pere Toran, Marta Trapero-Bertran, Iris Lozano, Silvia De Sanjose

**Affiliations:** 1 Atenció a la Salut Sexual i Reproductiva (ASSIR), SAP Cerdanyola –Ripollet, Institut Català de la Salut, Carretera N-150 s/n, Ripollet, Barcelona, Spain; 2 Unitat de Suport a la Recerca Metropolitana Nord. IDIAP Jordi Gol, Sabadell, Barcelona, Spain; 3 Universitat Autònoma de Barcelona, Bellaterra, Barcelona, Spain; 4 GRASSIR research group. IDIAP Jordi Gol. Generalitat de Catalunya, Barcelona, Spain; 5 Departament de Infermeria, Universitat Autonoma de Barcelona, Bellaterra, Barcelona, Spain; 6 SAP Valles OccidentalInstitut Catala de la Salut, Sabadell, Barcelona, Spain; 7 Sistemes d’Informacia Sanitaria, SAP Vallès Occidental, Institut Catala de la Salut, Sabadell, Barcelona, Spain; 8 Center for Research in Economics and Health (CRES), Universitat Pompeu Fabra, Barcelona, Spain; 9 Unit of Infections and Cancer, Institut Catala d'Oncologia, L'Hospitalet de Llobregat, Barcelona, Spain; 10 CIBER Epidemiologia y Salud Publica, Madrid, Spain

**Keywords:** Clinical trial, Experimental study, Population coverage, Population screening, Cervical cancer, Cervical cytology

## Abstract

**Background:**

Cervical cancer is a frequently diagnosed cancer in women worldwide. Despite having easy preventive and therapeutic approaches, it is an important cause of mortality among women.

**Methods:**

The CRICERVA study is a cluster clinical trial which assigned one of three interventions to the target population registered in Cerdanyola, Barcelona. Among the 5,707 resident women aged 60 to 70 years in the study area, women with no record of cervical cytology over the last three years were selected. The study included four arms: three interventions all including a pre-assigned date for screening visit and i) personalized invitation letter; ii) adding to i) an informative leaflet; and, iii) in addition to ii) a personalized appointment reminder phone call, and iv) no specific action taken (control group). Participants were offered a personal interview about social-demographic characteristics and about screening attitudes. Cervical cytology and HPV DNA test (HC2) were offered as screening tests. In the case of screening positive in any of these tests, the women were followed up until a full diagnosis could be obtained. The effect size of each study arm was estimated as the absolute gain in coverage between the original coverage and the final coverage.

**Results:**

From the intervention groups (4,775 women), we identified 3,616 who were not appropriately screened, of which 2,560 women answered the trial call and 1,376 were amenable to screening. HPV was tested in 920 women and cervical cytology in all 1,376. Overall, there was an absolute gain in coverage of 28.8% in the intervention groups compared to 6% in the control group. Coverage increased from 51.2% to 76.0% in strategy i); from 47.4% to 79.0% in strategy ii) and from 44.5% to 74.6% in strategy iii). Lack of information about the relevance of screening was the most important factor for not attending the screening program.

**Conclusions:**

The study confirms that actively contacting women and including a date for a screening visit, notably increased participation in the screening program. Efforts to improve health education in preventative activities are warranted.

**Trial registration:**

Clinical Trials.gov Identifier NCT01373723. Registered 14 June 2011.

## Background

Cervical cancer is the second most frequently diagnosed cancer in women worldwide, with at least 500,000 new cases detected each year [1]. Despite having easy preventive and therapeutic approaches, this neoplasm continues to be an important cause of morbidity and mortality among women, particularly in developing countries where screening is infrequent or even absent.

In Spain, the age-adjusted (using world population age structure) incidence rate of invasive squamous cervical cancer is 6.3 cases per 100,000 women per year [1]. This incidence has remained constant during the 15 year period from 1983-1997, but an increase has been observed in the cohort of women born after 1930-1940, probably due to a lack of screening coverage [2]. The etiological cause of cervical cancer is infection by the human papillomavirus (HPV) [3]. Genotypes 16 and 18 are the most prevalent in cervical cancer cases within our setting and together with phenotypes 45, 31, 33, 35, 52, and 58 are responsible for 90% of the cervical cancers reported, with a global prevalence of 99.7% [4,5].

The Papanicolau test, which was introduced in the 1960s, led to a reduction of up to 80% in the incidence and mortality caused by this disease because it enables early diagnosis of pre-cancerous lesions [6] and it continues to be the principle diagnostic test used in screening programs worldwide. One of the limitations of this cytological test is its low sensitivity, and therefore periodic repetitions are needed. The detection of the HPV has been thoroughly researched in randomized clinical trials and is now being recommended as a primary screening tool in several screening programs [7].

In Spain screening for cervical cancer is largely opportunistic and cytology based [8]. In Catalonia the protocol for recommendations of preventive activities for cervical cancer was initiated in 2006 by the Directive Oncology Plan (Plan Director de Oncologia) and the Catalan Institute of Oncology (Institut Catala d‘Oncologia) [9]. In this region, there are only two studies that have evaluated the impact of screening among women with invasive cervical cancer. They reported that between 50% and 80% of women had not undergone previous cytology tests during the 10 years prior to cancer diagnosis [10,11]. Thus, an increase in screening coverage should be a priority objective for those responsible for health care policies, if the incidence of cervical cancer is to be reduced. This screening should identify and include those women who have not periodically had cytological tests. Some authors have reported that factors such as ethnic origin, age, education and socioeconomic level could influence participation in screening programs [12]. Five possible reasons for women to not undergo screening are: (a) the perception of vulnerability, (b) the benefits of screening are not perceived, (c) anxiety, (d) confusion, and (e) the fear of cancer, family difficulties or personal circumstances [12]. In terms of the factors influencing participation in screening, some authors have suggest the following reasons: the absence of population-based programs, low sensitization with respect to preventive attitudes in cohorts of elderly women and, health care overload in primary care centres [10,13].

In a systematic review from the Cochrane collaboration [12] evaluating interventions to stimulate women’s participation in screening programs for this disease, the authors concluded that invitations and educational interventions seem to be the most effective ways to increase participation. In addition, there is sufficient evidence to increase coverage by using individualized information directed at the target population [13,14]. There is strong evidence that call-recall systems (i.e. sms, email or phone calls) are effective as well (13). Forbes et al. encourages implementing trials such as the one presented here, to further support strategies to increase coverage [12].

The upper age limit for population screening is generally 65 years [9]. However, some studies [2,15,16] have observed an increase in the incidence of cancer in women born in the decade from 1930-1940. The Recommendations of the Spanish Consensus of 2006 on the secondary prevention of cervical cancer [2,16] and Resolution 287 of the European Council (June 10, 2008 Luxemburg), related to the volume of cancer in the European Union and the mechanisms to reduce this prevalence [15], recommend the age of screening be raised to 70 years.

The incidence of and mortality from cervical cancer are not uniform in terms of age. In the elderly age group [2] incidence and mortality tend to converge, demonstrating, on one hand, the worse prognosis of tumors at advanced ages, and on the other hand, the relative absence of early diagnoses. It is relevant to take into account that information campaigns on the prevention of cancer, and sexual education in schools were inconsistently carried out in Spain and in 80s and 90s of the last century. Therefore, there is a particular interest and need to perform studies in this age group.

The CRICERVA project [5] is a population study including women aged 30-70 years. The aim of this study was to determine which of three intervention strategies was the most effective in terms of screening coverage for cervical cancer. As a secondary objective we analyzed the factors associated with screening adherence and coverage.

Adherence was defined as a collaborative response to both the invitation from the physician to perform an interview and a medical visit according to appropriateness. In this study we only present data restricted to women aged from 60-70 years. Any women selected in this study with a confirmed history of not being appropriately screened for cervical cancer were offered a cervical cytology with an HPV DNA test, according to the standard recommendations.

## Methods

The CRICERVA study [5] is a multicenter, randomized, controlled, community-based cluster clinical trial, with four arms and performed in the SAP of Cerdanyola, Barcelona, Spain. It covers a population of 120,293 individuals over the age of 14 years. This study was approved by the Ethical Committee of the IDIAP Jordi Gol, and was registered at the Clinical Trials.gov identifier NCT01373723 on the14th of June in 2011.

All the women were adequately informed about the screening procedures and the significance of the results of both cervical cytology and HPV DNA tests when they came for the programmed visit. All participants signed an informed consent.

We targeted all women (N = 3,616) aged from 60 to 70 years of age, living in an area in which we could not retrieve any cytological test results from their medical records, or with a cytology test carried out more than 3 years before in women aged 60 to 65 years, or a previous cytology before the age of 60 if the women were 65 years old or more.

### Exclusion criteria

The following exclusion criteria were applied: (a) hysterectomized women with a history of pre-malignant lesions (AGUS ASCUS, LSIL, HSIL), carcinoma in situ or cervical-uterine cancer, HIV positive or other causes of immunosuppression (since these women follow a specific protocol); (b) non residents in the area for more than 6 months.

### Sample size

The sample size was calculated based on the detection of a difference in coverage among the intervention group compared with the non intervention group (NIG). It was calculated by multiplying the size of a simple randomized design by the design effect or inflation factor. For the simple randomized design, accepting an alpha risk of 0.05 and a beta risk of 0.20 in a bilateral contrast, 59 subjects were estimated to be required in the first group and 59 in the second group, to detect a difference greater than or equal to 28.4% in the screening coverage of the 41.6% in the NIG. The loss to follow up rate was estimated at 20%. The calculation of the sample was performed with the Granmo 5.2 computer program for Windows. According to a literature review [17,18] considering an intraclass correlation coefficient of 0.05 and a mean number of 3,500 women from 30 to 70 years of age with incorrect screening in each Basic Health Care Assistance (BHCA), the design effect was 176 and thus, 20,768 women who were not appropriately screened needed to be studied.

### Randomization process

The cluster unit was each BHCA to avoid a contamination risk among women in the close geographical areas. This study is based on the Public Primary Health Care Services SAP Cerdanyola together with the Sexual and Reproductive Health Program, provided free of charge. SAP Cerdanyola is divided into five BHCAs, 4 of which had similar socio-economic characteristics. One BHCA was excluded because it had a very high socio-economic status compared to the other 4 BHCA. Each BHCA was randomly assigned to each arm.

### Study arms

Each of the 4 participating centres was assigned to one study arm each:

1. NIG: includes women opportunistically attending the clinic. These women were entered into the routine protocol.

2. IG1(Intervention group 1): a personalized letter was sent to the participant and signed by the patient’s primary care physician and professionals from the corresponding Public Health Center.

3. IG2 (Intervention group 2): the same invitation letter as that used in the IG1 was sent to the participant, as well as an informative leaflet on the prevailing reasons for screening cervical cancer. We evaluated the repercussion of informed participation.

4. IG3 (Intervention group 3): the same intervention as the one performed in IG2, complemented by a phone call 3 days prior to the appointment date indicated in the invitation letter, as a reminder of the visit.

All the interventions arm received a pre-assigned date for the screening visit.

### Clinical effectiveness measures

The primary outcome measure was the percentage of women who accepted to be screened and were finally categorized as adequately screened. The secondary outcome measures included the total number of cytologies performed, HPV infections detected, different grades of cervical intraepithelial lesions detected and cancer detected. The follow up period of this trial finished when the diagnosis of each screening visit was completed.

### Information sources

All participants were given a structured questionnaire to obtain information related to sociodemographic, medical and behavioral factors including date of birth, country of origin, education, job situation, level of income, family income, marital status, number of children, care-giver to dependents, type of gynecological care and history of cervical-uterine disease. We also collected information on previous screening and, if so, type of medical assistance (private or public), frequency of visits, the result of the last cytology test performed and previous HPV tests. After having completed the recruitment of the intervention groups, all women identified in the control group characterized as having an incorrect screening were invited to answer the questionnaire via a telephone call. A review of the participants’ clinical history on the Information Systems (eCAP) of the area was also undertaken to obtain information related to clinical data.

### Statistical analysis

Qualitative variables are described with absolute frequencies and percentages. The quantitative variables are expressed with means and standard deviations. The Chi-square test was used in the qualitative variables comparison, and analysis of variance for the quantitative variables analysis. The effect size of each study arm was quantified as the difference in coverage before and after the intervention. The level of significance used was p ≤ 0.05. The analyses were performed with the SPSS statistical package for Windows v. 20.0.

## Results

### Participation and adherence

**Figure 1:**
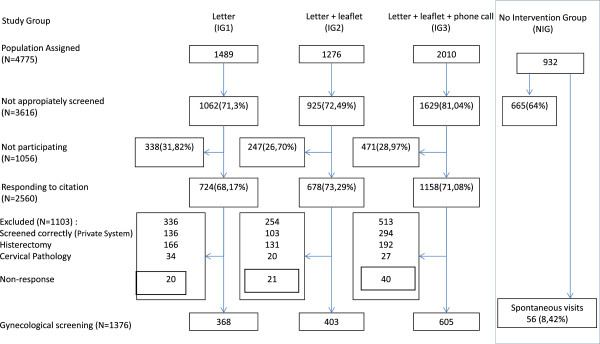
Flowchart of the population sample assigned to the centres and assigned to each of the intervention study arms, the population with no previous screening, the participation achieved and the final coverage obtained in each intervention arm.

A total of 5,820 women aged 60 to 70 years old enrolled in the Primary Health Care centers were included: 1,489 patients in IG1, 1,276 in IG2, 2,010 in IG3 and 932 in the NIG (Figure 1). The screening situation was unknown for 3,616 women in the intervention groups and 665 in the NIG. A response was obtained from 2,560 women after the intervention, representing an adherence of 70.8%. Appointments were attended by 724 (68.2%) women in IG1, 678 (73.3%) in IG2, and 1,158 (71.1%) women in IG3. In the NIG 56 women (8.4%) spontaneously requested a visit to perform the cytology test.

After identifying women who fulfilled the criteria of incorrect screening, cervical cancer screening was carried out in 1,376 women (368 from IG1, 403 from IG2 and 605 from IG3). A large proportion of women in the intervention groups answered the personal questionnaire during their gynaecological visit (GI173.3%, GI2 68.2% and GI3 71.1%) while in the control group the telephonic contact was possible in 52% of women identified with incorrect screening.

Table 1 describes the characteristics of the women who participated in the personalized interview. Overall, the average age of the women was 66.2 (SD 4.0) years, and most were of Spanish origin, with a primary education level (32.2%) or incomplete primary education level (34.9%), while 16.7% had not received any education and 3.1% reported university studies. Most women were married (70.8%) and had children (94.6%) and 36.1% of the women declared having a monthly family income of between 1,000 to 2,000 euros.

**Table 1:** Sociodemographic characteristics, screening history and reason for non attendance to screening by intervention activity of the study population

		**Intervention groups**				
**Characteristic**	**No intervention group (NIG)**	**Letter (IG1)**	**Letter + leaflet (IG2)**	**Letter + leaflet + phone call (IG3)**	**Total**	**P**
**Interviewed**	349	724	678	1158º	2909	
**Age mean (SD)**	66.6 (4,1)	66.3 (4,1)	66.1 (4.1)	66.1 (3,9)	66.2 (4.0)	0.029
**Spanish nationality**	340 (98.3%)	505 (96.4%)	514 (95.9%)	740 (96.0%)	2099 (96.4%)	<0.001
**Educational level**						
None	46 (13.4%)	99 (22.1%)	81 (18,1%)	95 (14,0%)	321 (16.7%)	<0.001
Incomplete Primary	128 (37.2%)	177 (39.5%)	138 (30,8%)	226 (33,4%)	669 (34.9%)	
Primary	110 (32.0%)	130 (29.0%)	151 (33,7%)	226 (33,4%)	617 (32.2%)	
High School	54 (15.7%)	35 (7.8%)	58 (12,9%)	103 (15,2%)	250 (13%)	
University	6 (1.7%)	7 (1.6%)	20 (4,5%)	27 (4,0%)	60 (3.1%)	
**Monthly family income**						
from 0 to 600€	76 (30.3)	103 (26.6)	80 (21.4)	94 (16,3)	353 (22.3)	<0.001
from 601 to 1000 €	123 (49.0)	126 (32.6)	109 (29.2)	166 (28,9)	524 (33.0)	
from 1001 to 2000€	49 (19.5)	137 (35.4)	149 (39.9)	238 (41.4)	573 (36.1)	
more than 2000€	3 (1.2)	21 (5.4)	35 (9.4)	77 (13.4)	136 (8.6)	
**Marital status-married**	211 (60.6%)	340 (74.9%)	325 (72.9%)	489 (72.0%)	1365 (70.8%)	<0.001
**Number of children**						
0	13 (3.7%)	26 (5.7%)	29 (6.5%)	36 (5.3%)	104 (5.4%)	<0.001
1-2	132 (37.8%)	242 (52.7%)	222 (49.4%)	362 (53.4%)	958 (49.5%)	
>2	204 (58.5%)	191 (41.6%)	198 (44.1%)	280 (41.3%)	873 (45.1%)	
**Type of gynecological care**						
Public	231 (66.8%)	216 (48.0%)	232 (50.7%)	301 (45.1%)	980 (51.0%)	<0.001
Private	49 (14.2%)	126 (28.0%)	119 (26.0%)	235 (35.2%)	529 (27.5%)	
Mixed	4 (1.2%)	13 (2.9%)	33 (7.2%)	22 (3.3%)	72 (3.7%)	
None	62 (17.9%)	95 (21.1%)	74 (16.2%)	110 (16.5%)	341 (17.7%)	
**Lag time since last Pap screening**						
1-3	136 (39%)	208 (46.6%)	192 (45.1%)	340 (50.1%)	876 (46.1%)	<0.001
4-6	146 (41.8%)	131 (29.4%)	134 (31.5%)	194 (28.6%)	605 (31.9%)	
never	67 (19.2%)	107 (24.0%)	100 (23.5%)	144 (21.2%)	418 (22.0%)	
**Reasons for non-attendance to screening for women with no previous Pap**						
Fear and dislike	4 (6.0%)	43 (42.2%)	42 (42.4%)	50 (37.0%)	139 (34.5%)	<0.001
Uninformed	63 (94.0%)	56 (54.9%)	53 (53.5%)	84 (62.2%)	256 (63.5%)	
Other	0 (0.0%)	3 (2.9%)	4 (4.1%)	1 (0.7%)	8 (2.0%)	

Although most of the participants in all groups reported use of public health care centers for screening visits (51%), the use of private centres was not homogeneous among the groups, and was particularly low in the control group (14%) compared to overall (27%).

No type of gynecological care was reported in 17.7% of all the women. Out of all the women interviewed, 22% reported no previous cytology and 31.9% stated that the last cytology was 4 to 6 years before the interview. In almost half of the interviews (46.1%) a previous cytology test was performed within an interval of ≥3 years, although this percentage was significantly higher in IG3 (50.1%), (p < 0.001). Out of the women reporting no previous screening cytology tests, 63.5% indicated that the reason for this was lack of knowledge on its importance and 34.5% reported being afraid or disliking the test. The analysis of the medical records in the target population identified population coverage of 41.6% through the public health system. After obtaining the information from the women themselves, cervical cancer screening coverage prior to our intervention was estimated to be 47.4% (Table 2). This information was unknown for the control group.

**Table 2:** Target population and participation by intervention activity

**Characteristic**	**Letter (IG1)**	**Letter + leaflet (IG2)**	**Letter + leaflet + phone call (IG3)**	**Total intervention groups**
Assigned population	n = 1489	n = 1276	n = 2010	n = 4775
Initial coverage (*)	763 (51.2%)	605(47.4%)	894(44.5%)	2262(47.4%)
Final coverage	1131 (76.0%)	1008 (79.0%)	1499 (74.6%)	3638(76.2%)
Difference	368 (24.7%)	403(31.6%)	605 (30.1%)	1376 (28.8%)
95% CI	(22.5-26.9%)	(29.0 - 34.1%)	(28.1 - 32.1%)	(27.5 - 30.1%)

Initial coverage: Women with previous screening in computerized registries plus those reported to have been correctly screened or presenting reasons for exclusion (hysterectomy, change in residence, disease at the time of the visit).

CI: Confidence Interval.

### Increase in coverage after the intervention

After carrying out the interventions in each arm, the global coverage increased up to 76.2% (with a range between groups of 74.6% to 79.0%). Thus, the coverage rose by 24.7% in IG1, by 31.6% in IG2 and by 30.1% in IG3. The difference between IG3 and IG2 was very minor (1.5%) and more marked (7%) between IG1 and IG2.

Since only 52.5% of the control group answered the questionnaire about screening practices our estimates on the real coverage could not be fully estimated.

### Cytology results and detection of HPV

A total of 1,376 cytology tests and 920 HPV determinations (HC2) were performed in the intervention groups, with 99.3% showing negative results. Fifty-five cytology tests were carried out in the control group, all with negative results.

Of the 9 positive cytology results, 2 suspected cancers were detected and histologically confirmed, 3 High Squamous Intraepithelial Lesion (HSIL) and 4 Atypical Squamous Cervical Under Signification (ASC-US).

Twenty-two women were found to be HPV positive, of which 13 (59.1%) presented negative cytology results. All the abnormal cytologies were detected in HPV positive women.

## Discussion

This study confirmed that an active search for women to undergo screening for cervical cancer considerably increased participation rates. According to our results, this preventive intervention avoided several cases of severe disease in the population. We decided to use a letter with a fixed appointment date for the three study arms because it has been endorsed by different studies [12], and we could compare this intervention with other screening strategies as well as with the control group. We observed that the 3 strategies calling for screening significantly increased the participation from 47.4% up to 76.2%. The greatest increase in coverage was obtained in IG2 (letter + leaflet) compared to IG1 (only the letter). The addition of a phone call hardly improved the percentage of coverage obtained in the IG2.

While among women in the NIG the level of participation was only 8.4%, all other intervention groups showed participation levels greater than 68%. This confirms that a direct intervention, even at advanced ages, increases the adherence to screening.

Our study is in agreement with previous evaluations [12,19] supporting that coverage can easily be increased when there is individual contact with each woman. Although this approach implies organizing screening activities, in the long run this will have a major impact on women’s health. In our study, adherence was higher than that observed in other studies aiming to increase coverage using similar types of interventions and in similar age groups [14]. We do not yet know if these data will be similar in younger age groups. This work is still in progress.

It is important to note that the letters were sent by the family physician and the coordinators of the Public Health Center who in general were professionals known by the target population. In the letter it was indicated that the screening visit would be performed by a woman and the time and day of the appointment was specified, thereby facilitating the subsequent visit [13].

Different studies have reported that the key question influencing participation is the precision of the registries [12,13]. The tools used in the present study were the eCAP and the information system of the Catalan Public Health System which is continuously updated.

Our intervention not only increased screening coverage but also, with the prevailing protocol, allowed women who were not appropriately screened for cervical cancer to benefit from a co-test with cervical cytology and HPV. Both tests provided a very high negative predictive value for women with negative results thereby allowing these women to avoid being called up for posterior cervical screening. In contrast, 9 cases of CIN2+ were detected, 2 undergoing a hysterectomy for grade 1 cancer. If these women had not been identified, their cancer could have evolved to a more advanced invasive form of the disease. Thus these data although based on a limited number of observations, allowed the identification of 100-fold more pathology than what would have been expected in the general population of the same age [14], where the incidence of invasive cervical cancer is estimated to be 7.3 per 100,000 (1).

A low detection of HPV was confirmed in the elderly women. This result is similar to those obtained in other studies [20] confirming that women of this age belong to highly monogamous cohorts or have had time to eliminate the potential infection. In this elderly cohort, all pathological cytologies were also HPV positive. In addition, 13 women were HPV positive but with a negative cytology and remain under surveillance.These data indicate that for women aged 60 to 70, screening by the HPV and cervical cytology as a triage test is a very good strategy for capturing CIN2+ lesions (through positive results). At present, the protocol of the Department of Health of the Generalitat of Catalonia recommends the use of co-testing with cytology and HPV in women over 39 years who are not appropriately screened. Considering the results obtained in the present study, if primary screening was based on HPV detection and cytology as a triage test, only 19 cytologies would have been necessary in addition to the HPV tests. If the results of HPV are negative the screening controls of this elderly cohort should be finalized.

There were some limitations when interpreting our data. One limitation is that private medical care is rarely registered in the public health system although it is recommended therefore the information on the private medical practice was obtained through personal interviews but was not verified. Another limitation is that the control group was interviewed via telephone and the response rate was significantly lower compared to the intervention groups. This difference could affect the comparison of screening coverage prior to intervention. Moreover it could be speculated that individual randomization could have been a better option to control for co-factors affecting screening uptake. However, the geographical areas selected for randomization were chosen to avoid ‘contamination’ between our intervention groups by clearly separating them geographically. Finally, it was surprising that the introduction of a telephone call did not substantially increase coverage. Although we had several trained call operators a reluctance to answer the phone or to accept a short conversation seems to be a major obstacle, due to the overuse of this communication pathway by commercial purposes.

## Conclusions

Our cervical cancer intervention study among inappropriately screened women found that: 1) actively arranging appointments through a letter with a pre-assigned medical visit significantly increased participation rates, 2) including an informative leaflet with the letter achieved the greatest level of participation as well as a greater final coverage, and 3) a lack of information was the main reason for not attending screening visits.

## Abbreviations

ASC-US: Atypical squamous cervical under signification; ASSIR: Sexual and reproductive health care; BHCA: Basic health care assistance; CIN: Cervical intraepithelial Neoplasia; eCAP: Electronic clinical data base system for primary health care; HC2: Hybrid capture 2; HPV: Human papillomavirus; HPV DNA test: Deoxyribonucleic acid; HSIL: High squamous intraepithelial lesion; IDIAP Jordi Gol: University institute in primary care research Jordi Gol; IG: Intervention group; LSIL: Low grade squamous intraepithelial; NIG: Non intervention group; SAP: Primary health care service.

## Competing interests

The authors declare that they have no competing interests.

## Authors’ contributions

AA, JMM, DR, AR, JMB, NS and PS collaborated carrying out the study. AA, JMM. SDS contributed in the writing and preparation of the present manuscript. AA, JMM, SDS, DR, JMB, PH, PS, PT, MT, IL contributed in the preparation and revision of the present manuscript. All the authors have revised and approved the present manuscript. AA participated in the design, coordination and execution of the study, interpretation of data, writing of the manuscript and supervision of the project. This article will be used in his doctoral thesis in the Department of Medicine, Universitat Autonoma de Barcelona (UAB). JMM participated in the design, execution of the study, in the analysis and interpretation of data, critical revision of the manuscript and approval of the final draft. DR, JMB, PH, PS, PT, MT, IL participated in the research team, contributed to the study design, execution of the study, interpretation of data, critical revision of the manuscript and approval of the final draft. NS participated in the research team, contributed to the study design, execution, critical revision of the manuscript and approval of the final draft. SDS participated in the research team, contributed to the interpretation of data, critical revision of the manuscript and approval of the final draft.

## Pre-publication history

The pre-publication history for this paper can be accessed here:

http://www.biomedcentral.com/1472-6874/14/86/prepub
